# Pan-Cancer Analysis of the Oncogenic and Prognostic Role of PKM2: A Potential Target for Survival and Immunotherapy

**DOI:** 10.1155/2023/3375109

**Published:** 2023-02-21

**Authors:** Nan Liang, Lusi Mi, Jingting Li, Tong Li, Jun Chen, Gianlorenzo Dionigi, Haixia Guan, Hui Sun

**Affiliations:** ^1^Division of Thyroid Surgery, The China-Japan Union Hospital of Jilin University, Jilin Provincial Key Laboratory of Surgical Translational Medicine, Jilin Provincial Precision Medicine Laboratory of Molecular Biology and Translational Medicine on Differentiated Thyroid Carcinoma, Changchun City, Jilin Province, China; ^2^Division of General and Endocrine Surgery, Istituto Auxologico Italiano IRCCS, Department of Medical Biotechnology and Translational Medicine, University of Milan, Milan, Italy; ^3^Department of Endocrinology, Guangdong Provincial People's Hospital (Guangdong Academy of Medical Sciences), Southern Medical University, 106 Zhongshan Er Road, Guangzhou, 510080, China

## Abstract

**Background:**

No pan-cancer study has been conducted till date to explore the comprehensive oncogenic roles of pyruvate kinase M2 (PKM2).

**Methods:**

TCGA, TIMER, GEPIA, UALCAN, STRING, and other databases were used to analyze the expression, prognostic roles, epigenetic variants, and possible oncogenic mechanisms of PKM2. Proteomic sequencing data and PRM were applied to validate.

**Results:**

PKM2 showed higher expression in majority of cancers, the expression being significantly correlated with the clinical stage. Higher expression of PKM2 was associated with lower OS and DFS in several cancers, such as MESO and PAAD. In addition, the epigenetic variation of PKM2, including gene alteration, mutation type and sites, DNA methylation, and phosphorylation, showed diversity in different cancers. All four methods indicated that PKM2 is positively associated with the immune infiltration of tumor-associated fibroblasts, such as in THCA, GBM, and SARC. Further mechanistic exploration suggested that the ribosome pathway might play an essential role in the regulation of PKM2, and interestingly, four out of ten hub genes were found to be highly related to OS in several cancers. Finally, in thyroid cancer specimen, we validated the expression and potential mechanisms by proteomic sequencing and PRM validation.

**Conclusion:**

In the majority of cancers, the higher expression of PKM2 was highly associated with poor prognosis. Further molecular mechanism exploration implied that PKM2 might serve as a potential target for cancer survival and immunotherapy by regulating the ribosome pathway.

## 1. Introduction

Tumors are the biggest public health problem worldwide [1]. Investigation of the mechanisms of tumorigenesis and search for potential prognostic biomarkers of tumors are extremely essential. Pyruvate kinase M2 (PKM2) is a key enzyme of the glycolytic pathway that catalyzes the transfer of phosphate groups from phosphoenolpyruvate (PEP) to ADP in order to generate ATP [2, 3]. PKM1 and PKM2 are encoded by the PKM gene. Mutually exclusive selective splicing of exons 9 and 10 in mammals gives rise to PKM1 containing exon 9 and PKM2 containing exon 10, respectively [4]. PKM2 exists in two forms, namely, a highly active tetramer and a relatively less active dimer. The ratio between the two forms determines whether carbon from glucose would be directed into the biosynthetic process or be used for glycolytic ATP production. The transition between the two forms is crucial not only for the control of glycolysis but also for the proliferation and survival of tumor cells [2–7]. PKM2 has been reported to be highly expressed in a variety of tumors, and dominant high expression of the low-activity dimeric PKM2 isoform is thought to be critical for aerobic glycolysis and tumor growth in tumor cells [8]. The current study was aimed at investigating the cross-cancer oncogenic role and prognostic value of PKM2 by evaluating multiple databases.

## 2. Materials and Methods

### 2.1. Gene and Protein Expression and Survival Analysis

All the comprehensive data on cancers, including genomic, proteomic, transcriptomic, epigenomic, and clinical data, were obtained from The Cancer Genome Atlas (TCGA) database (https://www.cancer.gov/). TIMER2 (http://timer.cistrome.org/) was applied to analyze TCGA data for various cancer types [9]. The University of Alabama Cancer database (UALCAN) (http://ualcan.path.uab.edu/) was used to analyze the cancer OMICS data, using protein expression analysis data from the Clinical Proteomic Tumor Analysis Consortium (CPTAC) [10, 11]. All human protein-related information, either in cells, tissues, or organs, was obtained from Human Protein Atlas (HPA, https://www.proteinatlas.org/) and by integrating various omics technologies, including antibody-based imaging, mass spectrometry-based proteomics, transcriptomics, and systems biology [12]. GEPIA and GEPIA2 (http://gepia.cancer-pku.cn/, http://gepia2.cancer-pku.cn/) were used to explore the possible associations of PKM2 with different types of pathological cancer stages. Moreover, GEPIA2 analyzed the association between PKM2 expression and survival. The cutoff-high and cutoff-low were selected as 50% each, and log-rank *P* was used as the test value to obtain the corresponding results of “survival analysis.”

### 2.2. Epigenetic Changes

CBioPortal (http://www.cbioportal.org/) was applied to explore the genetic mutation analysis of tumors in TCGA database [13, 14]. MethSurv (https://biit.cs.ut.ee/methsurv/) was used for univariable and multivariable survival analysis based on DNA methylation biomarkers using TCGA data [15]. UALCAN was applied to analyze the protein phosphorylation information for a total of six tumors. A statistical significance of *P* < 0.05 was considered for the protein phosphorylation analysis.

### 2.3. Immune Infiltration Analysis

TIMER2 was used to investigate the potential link between gene expression and tumor immune infiltration, along with TIMER, CIBERSORT, CIBERSORT-ABS, QUANTISEQ, XCELL, MCPCOUNTER, and EPIC algorithms. *P* values and partial correlation (cor) values were accessed via purity-adjusted Spearman's rank correlation test [9].

### 2.4. Enrichment Analysis of PKM2-Related Genes

STRING (https://string-db.org/) enabled the construction of protein–protein interaction networks, which interacted with PKM2. GEPIA2 was used to explore similar genes as PKM2, and Venn diagrams (http://bioinformatics.psb.ugent.be/webtools/Venn/) were applied to analyze the genes that bind to and are associated with PKM2. Two sets of genes were uploaded to DAVID (https://david.ncifcrf.gov/) for GO enrichment and KEGG pathway analysis. The above-mentioned data were presented visually in HiPlot (https://hiplot.com.cn/). Kyoto Encyclopedia of Genes and Genomes (KEGG, https://www.kegg.jp/kegg/) was applied to analyze pathways enriched in PKM2-related genes, which was a database resource for understanding high-level functions and utilities of the biological system, such as the cell, organism, and ecosystem, from molecular-level information, especially large-scale molecular datasets generated by genome sequencing and other high-throughput experimental technologies [16]. Cytoscape was searched for hub genes from the genes interacting with PKM2; this enabled the integration of biomolecular interaction networks with high-throughput expression data and other molecular states into a unified conceptual framework [17].

### 2.5. Validation of PKM2 in THCA

MEXPRESS (https://mexpress.be/) is a data visualization tool designed for the easy visualization of TCGA expression, DNA methylation, and clinical data, as well as the relationships across them [18]. Thyroid cancer specimen and adjacent normal tissue were collected in the Department of Thyroid Surgery, China-Japan Union Hospital of Jilin University. Specimens were subjected to iTRAQ/TMT-labeled proteomic sequencing and PRM validation.

## 3. Results

### 3.1. Pan-Cancer Expression Profiles and Prognostic Roles of PKM2

**Figure 1:**
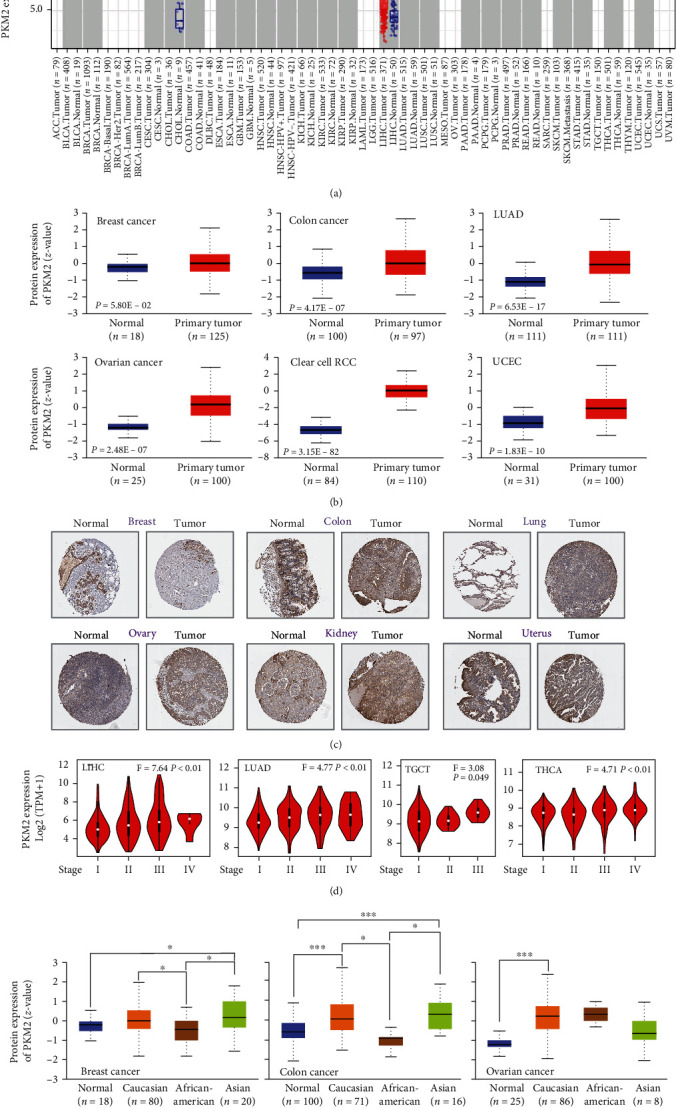
The pan-cancer expression profiles of PKM2. (a) The expression status of the PKM2 gene in different cancers or specific cancer subtypes. (b) The expression level of PKM total protein between normal tissue and primary tissue of breast cancer, colon cancer, lung adenocarcinoma, ovarian cancer, clear cell RCC, and UCEC. (c) Immunohistochemical results of PKM2 in a variety of tumors. (d) Correlation between PKM2 expression and pathological stage of LIHC LUAD, TGCT, and THCA. (e) The expression of PKM2 among different races. ^∗^*P* < 0.05;  ^∗∗^*P* < 0.01;  ^∗∗∗^*P* < 0.001.

**Figure 2:**
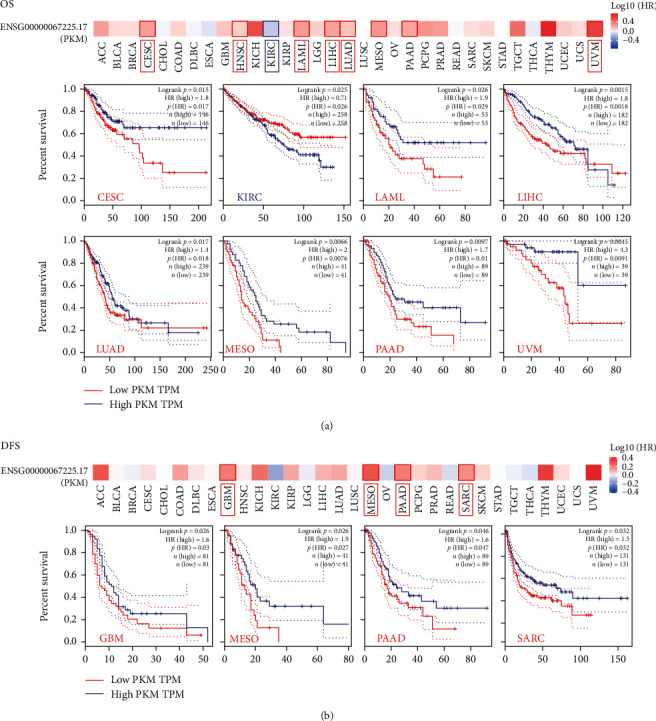
The pan-cancer prognostic roles of PKM2. (a) The overall survival. (b) The disease-free survival. The survival map and Kaplan-Meier curves with positive results are given.

First, the mRNA level of PKM2 in different cancers was explored from TCGA database (Figure 1(a)). Compared to the corresponding control tissues, the majority of tumors, including BLCA, BRCA, CHOL, and COAD, showed higher expression of PKM2. The protein level of PKM2 in cancers was also explored from the CPTAC dataset. Compared to that in the corresponding normal tissues, PKM2 showed higher expression in colorectal cancer, lung adenocarcinoma, ovarian cancer, clear cell RCC, and UCEC (Figure 1(b)). As shown in Figure 1(c) and Figure S1, the immunohistochemical images of PKM2 also suggested higher expression in cancers. The association between PKM2 expression and cancer stages was analyzed next. Only four types of cancers, namely, LIHC, LUAD, TGCT, and THCA, were found to show a significant association of their stage with PKM2 expression (Figure 1(d) and Figure S2). Besides, the differential expression of PKM2 among various races was explored. The results showed that in breast cancer, PKM2 expression was significantly higher in Asians than in African-Americans. And PKM2 expression was significantly lower in African-Americans than in Asians and Caucasians in colorectal cancer (Figure 1(e)). In addition, we paid special attention to prognostic roles of PKM2 in cancer (Figure 2 and Figure S3). PKM2 expression was highly associated with the overall survival in nine types of cancers, such as CESC, HNSC, KIRC, LAML, LIHC, LUAD, MESO, PAAD, and UVM. Except for that in KIRC, the higher expression of PKM2 was obviously associated with lower overall survival in the remaining eight cancers (Figure 2(a)). Similarly, higher expression of PKM2 was significantly associated with lower DFS in GBM, MESO, PAAD, and SARC (Figure 2(b)). Especially, PKM2 was related to both OS and DFS in MESO as well as PAAD. The obtained data suggested that PKM2 might play important roles in cancers.

### 3.2. Pan-Cancer Epigenetic Variations of PKM2

**Figure 3:**
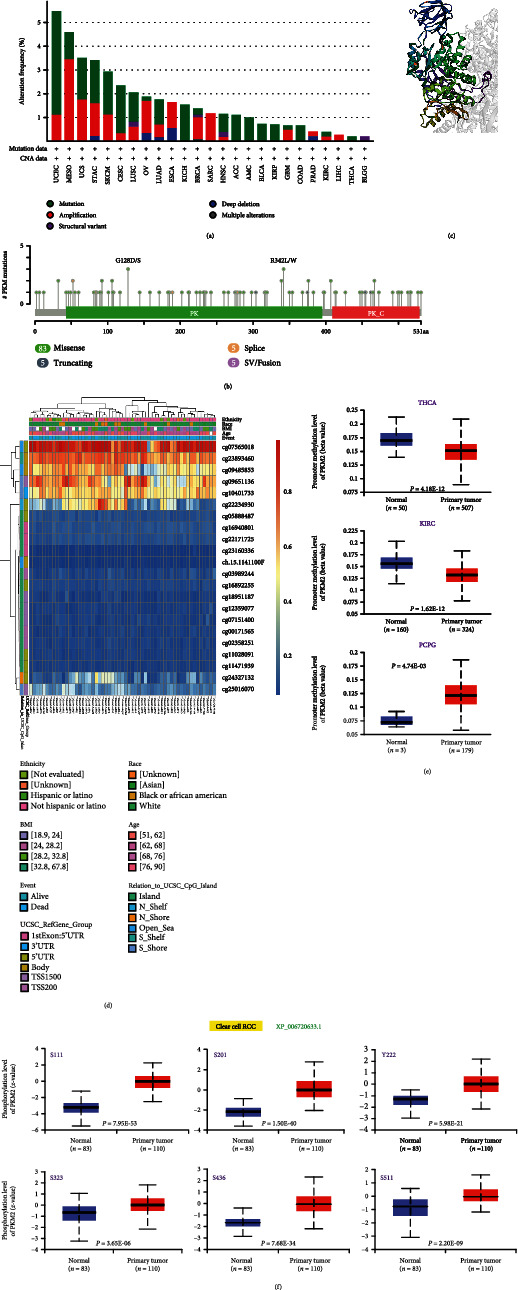
Epigenetic variants of PKM in different cancers of TCGA. (a) The alteration frequency and the mutation type. (b) The mutation site. (c) The 3D structure of PKM2. (d) The different methylation regions associated with PKM2 in UCEC. (e) The partial positive results of PKM2 promoter methylation levels in multiple tumors. (f) The expression level of PKM2 phosphoprotein (XP_006720633.1, S111, S201, Y222, S323, S436, and S511) in clear cell RCC. (e) The expression level of PKM2 phosphoprotein (NP_006720633, S111, S276, and S323) in UCEC.

To explore the potential functions of epigenetic variation of PKM2, we focused on its pan-cancer genetic alteration, DNA methylation, and phosphorylation. First, we observed the genetic alterations of PKM2 in different cancer samples of TCGA cohorts. As shown in Figure 3(a), in most cancers, the mutation was the predominant form of genetic alteration in PKM2. Moreover, the frequency of mutations was the highest in UCEC (>3%). In MESO, amplification was the predominant form of genetic alteration, accounting for more than 3% (Figure 3(a)). The types, locations, and the number of cases of genetic alterations of PKM2 are shown in Figure 3(b). Missense mutations were mainly concentrated in the domain PK, with R342L/W and G128D/S being the major mutation types. In addition, the three-dimensional view showed the spatial structure better (Figure 3(c)). Correlation between PKM2 genetic alterations and clinical survival, across patients with various tumors, was further investigated. In UCEC, OS and DSS were better in the genetically altered group, whereas DFS and PFS showed no significant difference (Figure S4).

PKM2 methylation was investigated next. As shown in Figure 3(e) and Figure S5, compared to the normal tissues, promoter methylation levels of PKM2 were significantly downregulated in the majority of cancers (except PCPG), such as BLCA, BRCA, CHOL, HNSC, KIRC, KIRP, PAAD, TGCT, PRAD, THCA, and UCEC. The heatmap in Figure 3(d) demonstrated the correlation of PKM2 methylation level of UCEC in different chips with clinicopathological features, such as race and BMI.

The expression levels of PKM2 phosphorylation between normal tissues and primary tumor tissues were compared based on the CPTAC database. Figures 3(d)–3(i) and Figure S6 summarize the PKM2 phosphorylation sites and their differences in six types of cancer. As shown in Table S2, in clear cell RCC and UCEC, the phosphorylation sites of PKM2 were mainly concentrated in the XP_006720633.1 while in NP_002645.3, PKM2 phosphorylation sites were fewer, especially in breast cancer and ovarian cancer. Among all the statistically significant results, only phosphorylation of PKM2 in colon cancer was downregulated at the S249 site compared to that in the normal tissue; however, this would require further investigation. The other genes were upregulated at the phosphorylation sites, especially in clear cell RCC, which showed significant phosphorylation across all of its sites (S111, S201, Y222, S323, S436, and S511). The potential functions and molecular mechanisms would need to be explored further.

### 3.3. Pan-Cancer Association between PKM2 Expression and the Level of Immune Cell Infiltration

**Figure 4:**
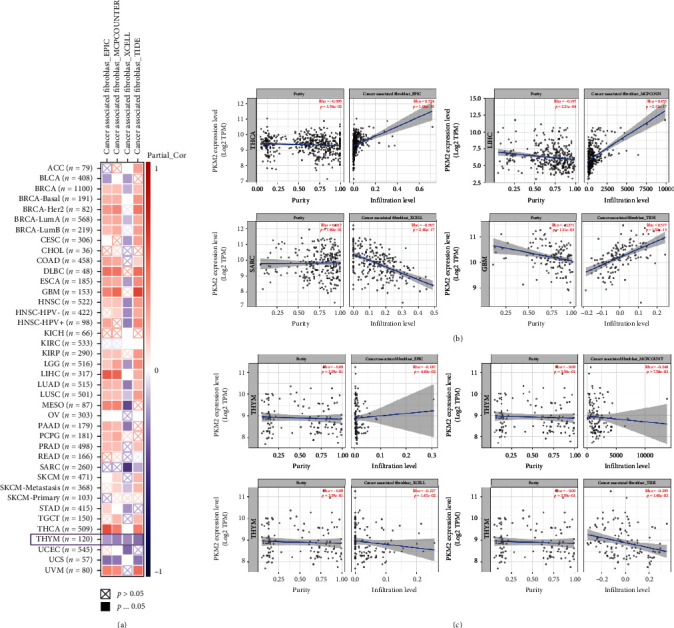
Correlation between PKM2 expression and tumor-associated fibroblast immune infiltration. (a) The potential correlation between the expression level of the PKM2 and the infiltration level of tumor-associated fibroblasts across all types of cancer. (b) The positive correlation between the expression level of the PKM2 and the infiltration level of tumor-associated fibroblasts. (c) The negative correlation between the expression level of the PKM2 and the infiltration level of tumor-associated fibroblasts in THYM.

The association between PKM2 expression and the level of different immune cell infiltration in various types of cancers was studied from TCGA using different algorithms, such as TIMER, CIBERSORT, CIBERSORT-abs, TIDE, XCELL, MCPCOUNTER, and EPIC. Interestingly, the immune infiltration of tumor-associated fibroblasts was negatively correlated with the expression of PKM2 when the XCELL algorithm was used (Figure 4(a)). At the same time, in THYM, a statistically negative correlation between the two was obtained, regardless of the algorithm used (Figure 4(c)). In contrast, when using the EPIC algorithm, PKM2 expression was found to be strongly positively correlated with fibroblasts in THCA (Figure 4(b)). Besides tumor-associated fibroblasts, we also found immune infiltration of B cells to be negatively correlated with the expression of PKM2 in the majority of tumors, including BRCA (Figure S7). The above results suggested a greater correlation between the expression of PKM2 and the level of tumor immune cell infiltration, which may be related to the occurrence and development of cancers. However, this interesting phenomenon would require further investigation.

### 3.4. Possible Molecular Regulatory Mechanisms of PKM2

**Figure 5:**
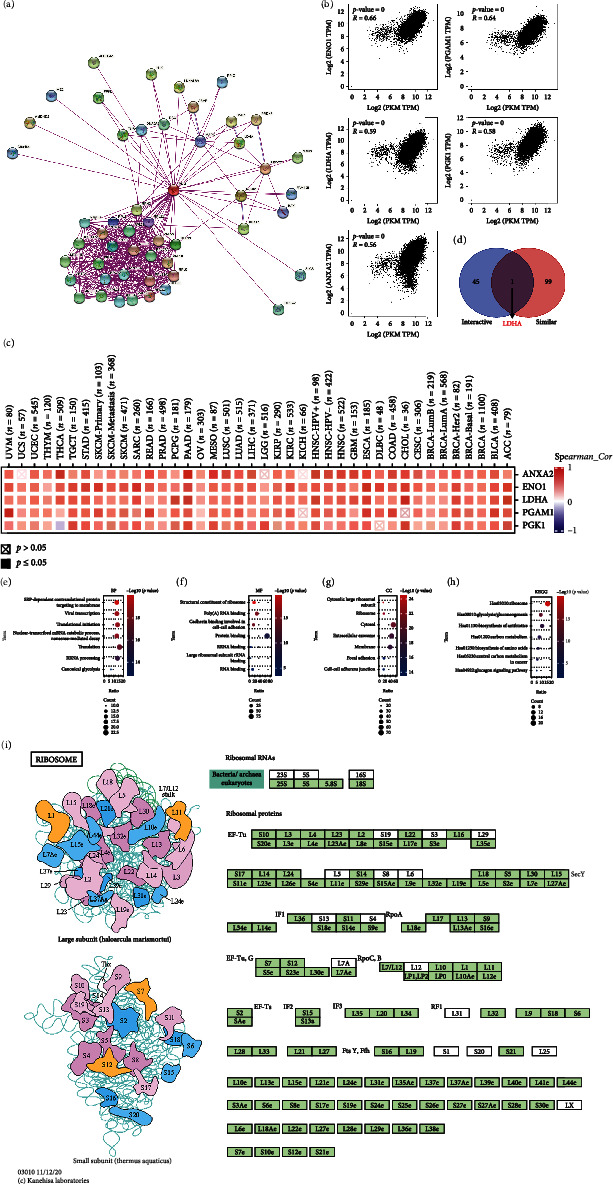
GO enrichment analysis of PKM2-related genes. (a) The network for PKM2 and the 46 interactive proteins. (b) The expression correlation between PKM2 and ENO1, PGAM1, LDHA, PGK1, and ANXA2 from the top 100 PKM2 similar genes. (c) The corresponding heatmap data in the detailed cancer types. (d) An intersection analysis of the PKM2 interactive and similar genes. GO enrichment analysis of target host genes based on three aspects, including (e) biological processes, (f) cellular components, and (g) molecular functions. (h) KEGG pathway analysis based on the PKM2 interactive and similar genes. (i) Structure diagram of ribosome.

**Figure 6:**
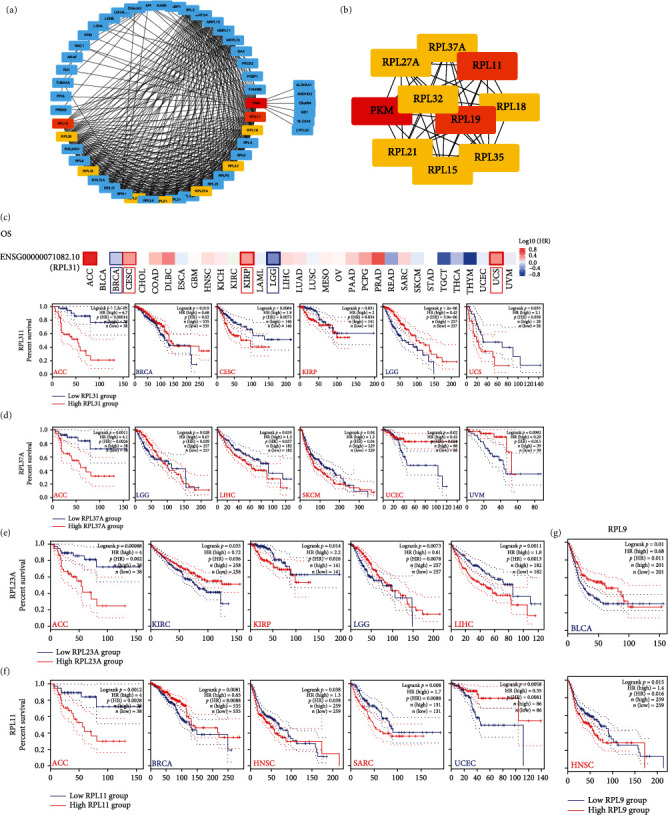
Detection and prognosis of hub genes from the PPI network. (a) The position of hub genes in PPI network. (b) The interaction between hub genes. (c) The correlations of the representative 10 Hub genes with OS.

Based on the interesting results discussed above, possible mechanisms of PKM2 expression were considered in order to determine its role in the development of cancers. Overall, “Ribosome” pathways were considered to be involved in the functional expression of PKM2. As shown in Figure 5(a), a protein-protein interaction network (PPI) was constructed using 46 proteins that interacted with PKM2, as supported by experimental evidence. Furthermore, combined with the GEPIA2 tool, expression of PKM2 was found to be positively correlated with that of ENO1, PGAM1, LDHA, PGK1, and ANXA2 (Figure 5(b)). The result was confirmed in Figure 5(c). Moreover, an intersection analysis of the PKM2-interacting and PKM2-correlated genes was conducted as shown in Figure 5(d). Common members of the two groups were LDHA. We performed GO enrichment with KEGG pathway analysis on all 145 genes from both the databases. Data suggested that the effect of PKM2 on tumor pathogenesis may be related to “SRP-dependent co-translational protein targeting to membrane” and “Viral transcription” in biological processes (Figure 5(e)). From the cellular component perspective, PKM2 may have affected “Cytosolic large ribosomal subunit” and “Ribosome,” leading to tumorigenesis (Figure 5(f)). The mechanism of PKM2 action in cancers may be related to “Structural constituent of ribosome” in terms of molecular function (Figure 5(g)). The above may have affected the “Ribosome,” “Glycolysis/Gluconeogenesis,” and “Biosynthesis of antibiotics” pathways, thus causing tumorigenesis (Figures 5(i) and 6(h)).

Coincidentally, in the previous sequencing data, the differentially expressed proteins in thyroid cancer were mainly enriched in the following pathways: ribosome, apoptosis, and glutathione metabolism. This suggested that PKM2 may play an oncogenic role by participating in the “Ribosome” pathway. Next, the top 10 hub genes in the PPI network were screened. Figures 6(a) and 6(b) display their interactions with each other. The top 10 hub genes, namely, RPL11, RPL19, RPL23, RPL5, RPL23A, RPL9, RPL31, RPL32, and RPL37A, were negatively correlated with PKM2 (Table S1) and were all ribosomal protein-related genes. This suggested once again that PKM2 is highly likely to be involved in the “Ribosome” pathway. We further detected the expression profiles of the ten hub genes across cancers (Figure S8). Most of them were highly expressed in cancers than in normal tissues. Importantly, as illustrated in Figures 6(c)–6(f) and Figure S9, hub genes showed association with OS in several cancers, suggesting their potential prognostic roles.

### 3.5. Validation of PKM2 in THCA

**Figure 7:**
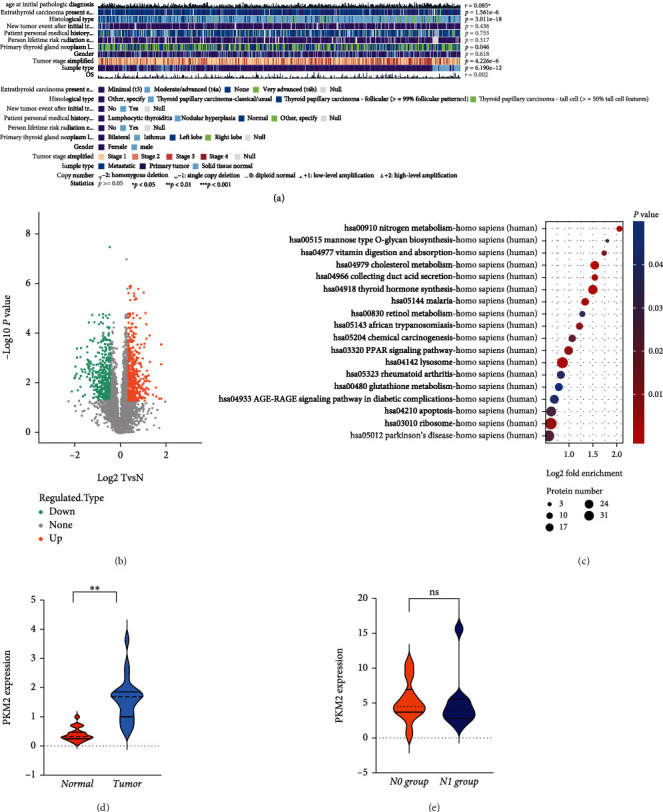
The expression and functional validation of PKM2 in THCA. (a) The correlation between PKM2 expression and clinicopathological characteristics of THCA patients in TCGA. (b) Differentially expressed proteins in normal thyroid tissue and tumor tissue. (c) Pathway enrichment analysis of differentially expressed proteins. (d) Differential expression of PKM2 protein levels in normal thyroid and tumor tissues. (e) The correlation between the expression of PKM2 and lymph node metastasis of thyroid cancer.

Among all the cancers, we first focused on the role of PKM2 in THCA. As shown in Figures 1(a) and 7(a), PKM2 was highly expressed in THCA, and PKM2 expression was associated with tumor stage, histological type, extra-thyroid carcinoma, and the diagnostic age. In some of our studies, six normal-tumor paired tissues were collected and further sequenced by iTRAQ-labeled LC-MS. A total of 5203 proteins were identified and quantified with the threshold fold change > 1.30 or <0.67. Compared to those in normal tissues, 487 proteins were significantly upregulated and 486 were significantly downregulated in tumor tissue. According to the proteomic data, PKM2 was indeed highly expressed in thyroid cancer (Figure 7(b)), and the differentially expressed proteins were mainly enriched in the following pathways: ribosome, apoptosis, and glutathione metabolism (Figure 7(c)). Additionally, another 20 paired specimens of THCA were collected and evaluated by PRM. As illustrated in Figure 7(d), PKM2 was further validated to have higher expression in thyroid cancer, compared to that in corresponding adjacent tissues. We analyzed the relationship between PKM2 expression and lymph node metastasis; however, we did not find a statistically significant relationship between the two (Figure 7(e)), possibly due to the small sample size. We hope to expand the sample size to continue with the validation in the future.

## 4. Discussion

In this study, we analyzed the pan-cancer expression, prognostic roles, epigenetic variants, and the possible oncogenic mechanism of PKM2, providing a theoretical foundation for the possibility of PKM2 as a pan-cancer marker.

PKM2 is a common isoform of pyruvate kinase. Prakasam et al. had classified PKM into four isoforms, including PKL, PKR, PKM1, and PKM2, based on their tissue distribution and selective gene splicing [19]. The expression of PKM2 in normal tissue cells is shown in Figure S10A-B. Figure S10C displays the position of PKM2 on the gene band. Several studies have shown that PKM2 is highly expressed in a number of tumor cells, such as colon cancer [20], non-small-cell lung cancer [21], gastric cancer [22], and melanoma [23]. In our present study as well, we obtained similar results. Further, we investigated the effect of PKM2 expression by survival analysis and found high PKM2 expression to be significantly associated with reduced OS. Lu et al. investigated the independent effect of PKM2 expression on survival status using univariate and multifactorial Cox regression analysis; they concluded that high PKM2 expression was significantly associated with reduced OS, and a worse prognosis was observed when comparing the PKM2-high expression group with the PKM2-low expression group [24]. Similarly, an article published in 2020 suggested that overexpression of PKM2 is associated with poor prognosis in patients with hepatocellular carcinoma [25].

Majority of current research suggests that epigenetic mechanisms mainly include DNA methylation, histone modifications, nucleosome remodeling, and RNA-mediated target [26, 27]. As shown in Figure 3, mutations in the R342L/W locus have missense concentrated in lung adenocarcinomas and uterine carcinomas; mutations in the G128D/S locus also have missense concentrated in acute myeloid leukemia, cutaneous melanomas, and squamous cell carcinomas of the lung. DNA methylation is an epigenetic process in which a methyl group is added to the cytosine base of DNA. This occurs most commonly at the CG dinucleotides, being referred to as CpG methylation. DNA methylation plays an important role in cell growth, differentiation, and disease development. Aberrant DNA methylation is often considered a characteristic feature of cancer development [28, 29]. Several studies have highlighted DNA methylation markers associated with differential cancer survival. For example, de Almeida et al. had identified novel DNA methylation markers in breast cancer, of which cg12374721 (PRAC2), cg18081940 (TDRD10), and cg04475027 (TMEM132C) are promising diagnostic and prognostic markers for breast cancer as well as others [30]. This phenomenon and its mechanism of formation need to be explored further and could potentially be a new target for targeted tumor therapy.

Phosphorylation is an important posttranslational modification of intracellular proteins. Existing studies have shown that PKM2 can acquire phosphate groups at specific sites under the action of protein kinases, leading to changes in activity and effects on tumor metabolism. Phosphorylation of PKM2 Y105, especially, has been widely reported in human cancers. In the experiments by Hitosugi et al., tyrosine phosphorylation was found to regulate PKM2 in order to provide tumor cells with a metabolic advantage, thereby promoting tumor growth [31]. However, in our current study, phosphorylation of PKM2 Y105 hardly played an important role in the development of multiple tumors. This phenomenon, therefore, should be explored further in the future.

Tumor-infiltrating immune cells, as an important component of the tumor microenvironment, are closely related to tumorigenesis, progression, or metastasis [32]. And we investigated the relationship between PKM2 expression and tumor-infiltrating immune cells represented by fibroblasts, B cells, T cell CD4+, T cell CD8+, NK cells, and mast cells. Results suggested that in some tumors, PKM2 expression may be associated with the degree of immune infiltration of tumor-associated fibroblasts. In addition, we integrated the information on PKM2 binding and PKM2 expression-related genes in all tumors, performed a series of enrichment analyses, and identified “ribosome,” “glycolysis/gluconeogenesis,” and “carbon metabolism” as possible influences in cancer etiology or pathogenesis.

Recently, numerous reports claiming the mechanism of regulation of tumor development by PKM2 have been published. For example, in a study by Zhu et al., unraveling of the specific molecular mechanisms of lncRNA-mediated PKM2 expression in cancer metabolism was reported [33]. Finally, we explored the PPI network for hub genes, namely, RPL11, RPL19, RPL23, RPL5, RPL23A, RPL9, RPL31, RPL32, and RPL37A. They were found to be associated with ribosomal proteins. This suggested that mutations in ribosomal proteins have the potential to cause tumorigenesis. In fact, mutations in ribosomal protein-coding genes have been reported in the literature in relation to T-cell acute lymphoblastic leukemia (RPL5 and RPL11) and gliomas (RPL5) [34]. In addition, although we have carried out the sequencing verification of PKM2 in thyroid cancer, it still needs to expand the sample size and conduct experiments to further verify the above conclusions.

In conclusion, PKM2 showed highly significant differential expression in a variety of tumors and was correlated with poor prognosis. In addition, further molecular mechanism exploration, including gene alteration, mutation type and sites, DNA methylation, phosphorylation, and GO enrichment, implied that Ribosome pathway might be involved in the functional expression of PKM2. Besides, immunological studies suggested PKM2 was positively associated with the immune infiltration. Those implied that PKM2 might serve as a potential target for cancer survival and immunotherapy by regulating the ribosome pathway.

## Data Availability

The data used to support the findings of this study are available from the corresponding author upon request.

## Supplementary Materials

Supplementary Materials Figure S1: immunohistochemistry images of PKM2 in various tumors and normal tissues, such as the liver, from HPA. These results suggested higher expression of PKM2 in tumors. Figure S2: the relationship between the expression levels of PKM2 and pathological stages of different tumors from TCGA and visualized by GEPIA2 tool. These results suggested that 19 types of tumors, such as COAD, did not show significant association of their stage with PKM2 expression. Figure S3: relationship between PKM2 pan-cancer expression and its survival progression from TCGA and visualized by GEPIA2 tool. These results suggested that a number of tumors did not show significant association of their OS with PKM2 expression, such as ACC. Figure S4: correlation between PKM2 genetic alterations and clinical survival prognosis in UCEC from TCGA and visualized by cBioPortal. It indicated that compared with altered group, unaltered group showed shorter disease-specific survival and progress free survival in UCEC. Figure S5: correlation between PKM2 expression and DNA methylation in multiple tumors from TCGA and visualized by UACLAN. These results suggested that except PCPG, PKM2 was significantly downregulated in majority of cancers, including BLCA, BRCA, CHOL, HNSC, KIRC, KIRP, PAAD, TGCT, PRAD, THCA, and UCEC. Figure S6: phosphorylation analysis of PKM2 protein in different tumors from CPTAC and visualized by UACLAN. (A-E) PKM2 phosphorylation sites and their differences in UCEC, LUAD, colon cancer, BRCA, and OV (positive results). (F-I) PKM2 phosphorylation sites and their differences in UCEC, LUAD, colon cancer, BRCA, and OV (negative results). Figure S7: correlation between PKM2 expression and immunocyte immune infiltration via TIMER2. (A) B cell. (B) T cell CD8+. (C) T cell CD4+. (D) NK cell. (E) Mast cell. These results suggested that immune infiltration of B cells to be negatively correlated with the expression of PKM2 in majority of tumors, including BRCA. Figure S8: the pan-cancer expression levels of Hub genes from TCGA and visualized by GEPIA2 tool, including RPL11, RPL19, RPL23, RPL5, RPL23A, RPL9, RPL31, RPL32, and RPL37A. These results suggested that most of them were highly expressed in cancers than in normal tissues. Figure S9: the pan-cancer correlations of the Hub genes with OS from TCGA and visualized by GEPIA2 tool. (A) RPL19. (B) RPL23. (C) RPL5. (D) RPL32. These results suggested that hub genes showed association with OS in several cancers, suggesting their potential prognostic roles. Figure S10: expression of PKM2 in normal tissues. (A) Expression of PKM2 in various types of normal tissues (RNA seq). (B) Expression of PKM2 in various types of normal tissues (Protein). (C) The location of PKM2 on Chr15. Table.S1: topological result exploration for top ten hub genes. These results suggested that the top 10 hub genes, namely, RPL11, RPL19, RPL23, RPL5, RPL23A, RPL9, RPL31, RPL32, and RPL37A, were negatively correlated with PKM2. Table.S2: sites of PKM2 protein phosphorylation. These results suggested that in clear cell RCC and UCEC, the phosphorylation sites of PKM2 were mainly concentrated in the XP_006720633.1 while in NP_002645.3, PKM2 phosphorylation sites were fewer, especially in breast cancer and ovarian cancer.
