# Supplementary material for: Pan-Cancer Analysis of the Oncogenic and Prognostic Role of PKM2: A Potential Target for Survival and Immunotherapy
Source: Biomed Res Int. 2023 Feb 21;2023:3375109. doi: 10.1155/2023/3375109 (PMC9974260; doi:10.1155/2023/3375109)
Supplement: Supplementary Materials — Figure S1: immunohistochemistry images of PKM2 in various tumors and normal tissues, such as the liver, from HPA. These results suggested higher expression of PKM2 in tumors. Figure S2: the relationship between the expression levels of PKM2 and pathological stages of different tumors from TCGA and visualized by GEPIA2 tool. These results suggested that 19 types of tumors, such as COAD, did not show significant association of their stage with PKM2 expression. Figure S3: relationship between PKM2 pan-cancer expression and its survival progression from TCGA and visualized by GEPIA2 tool. These results suggested that a number of tumors did not show significant association of their OS with PKM2 expression, such as ACC. Figure S4: correlation between PKM2 genetic alterations and clinical survival prognosis in UCEC from TCGA and visualized by cBioPortal. It indicated that compared with altered group, unaltered group showed shorter disease-specific survival and progress free survival in UCEC. Figure S5: correlation between PKM2 expression and DNA methylation in multiple tumors from TCGA and visualized by UACLAN. These results suggested that except PCPG, PKM2 was significantly downregulated in majority of cancers, including BLCA, BRCA, CHOL, HNSC, KIRC, KIRP, PAAD, TGCT, PRAD, THCA, and UCEC. Figure S6: phosphorylation analysis of PKM2 protein in different tumors from CPTAC and visualized by UACLAN. (A-E) PKM2 phosphorylation sites and their differences in UCEC, LUAD, colon cancer, BRCA, and OV (positive results). (F-I) PKM2 phosphorylation sites and their differences in UCEC, LUAD, colon cancer, BRCA, and OV (negative results). Figure S7: correlation between PKM2 expression and immunocyte immune infiltration via TIMER2. (A) B cell. (B) T cell CD8+. (C) T cell CD4+. (D) NK cell. (E) Mast cell. These results suggested that immune infiltration of B cells to be negatively correlated with the expression of PKM2 in majority of tumors, including BRCA. Figure S8: the pan-cancer exp [file 3375109.f1.zip › FigureS10.doc]

**Fig.S10.** Expression of PKM2 in normal tissues. A. Expression of PKM2 in various types of normal tissues (RNA seq). B. Expression of PKM2 in various types of normal tissues (Protein). C. The location of PKM2 on Chr15.
